# Supplementary material for: Changes in Plasma Lipid Levels Following Cortical Spreading Depolarization in a Transgenic Mouse Model of Familial Hemiplegic Migraine
Source: Metabolites. 2022 Mar 1;12(3):220. doi: 10.3390/metabo12030220 (PMC8953552; doi:10.3390/metabo12030220)
Supplement: Supplementary file 1 [file metabolites-12-00220-s001.zip › metabolites-1603668-supplementary.pdf]

metabolites MDPI

Supplementary Figures

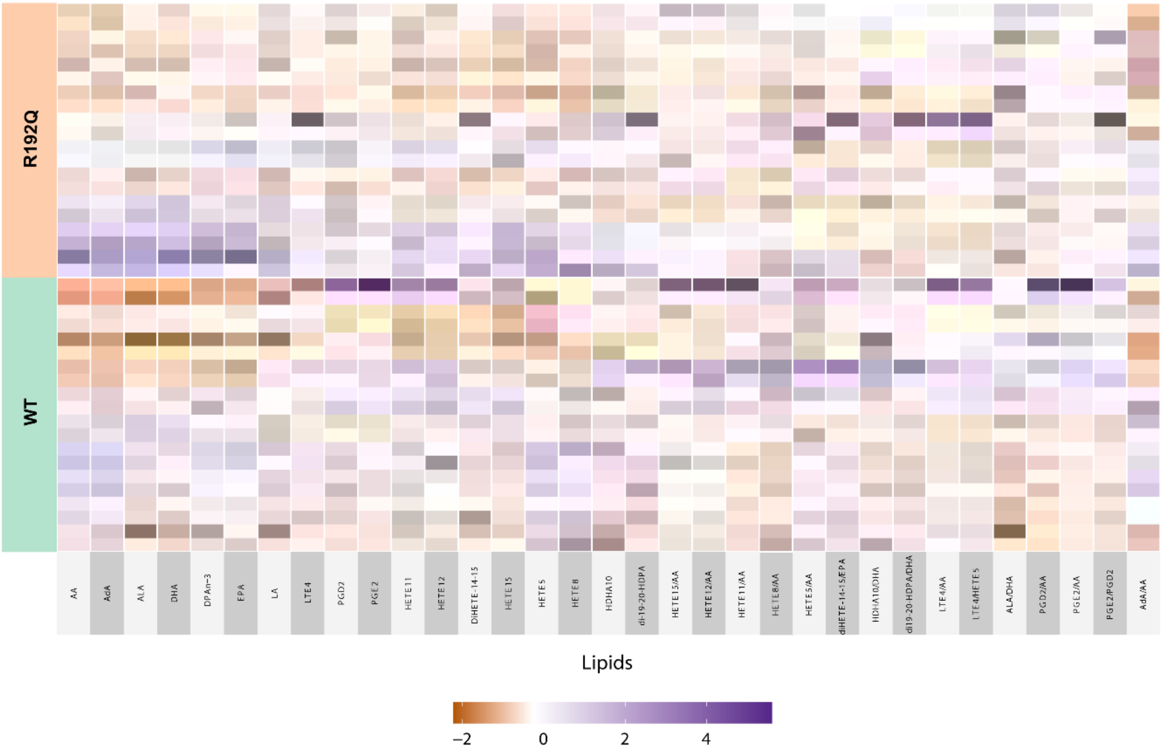

Figure S1. Heatmap summarizing the measured plasma lipid levels at baseline (per individual mouse) grouped by genotype. The map has no clear clustering structure, indicating that there are no pronounced differences in the concentration of measured lipids between WT and R192Q mutant mice.

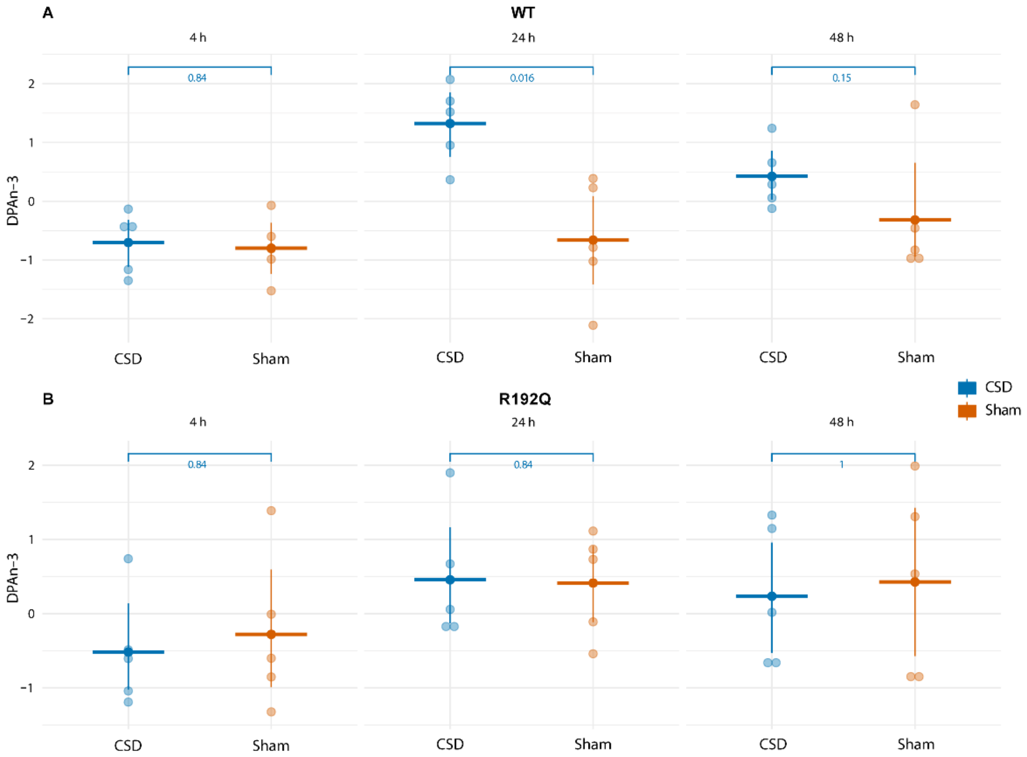

Figure S2. CSD is associated with long-term changes in DPAn-3 in WT mice. WT mice (A) showed an increase in lipid concentration of DPAn-3 at 24 h following CSD induction, that was no longer present at 48 h and not present in R192Q mutant mice (B).

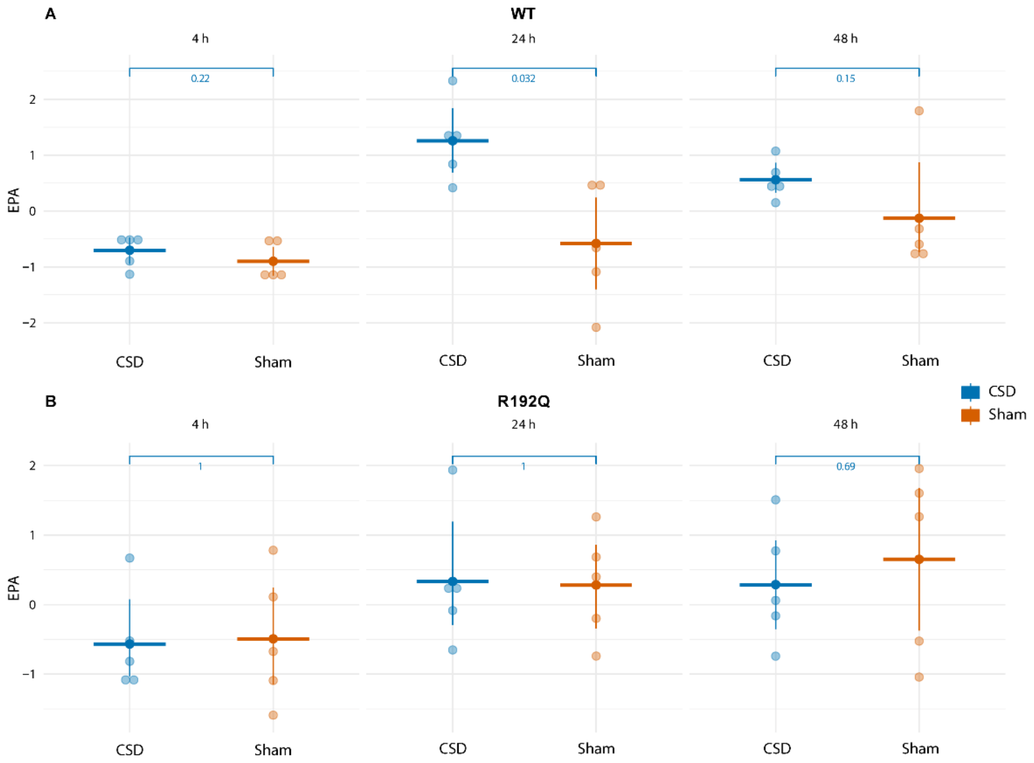

Figure S3. CSD is associated with long-term changes in EPA in WT mice. WT mice (A) showed an increase in lipid concentration of EPA at 24 h following CSD induction, that was no longer present at 48 h and not present in R192Q mutant mice (B).

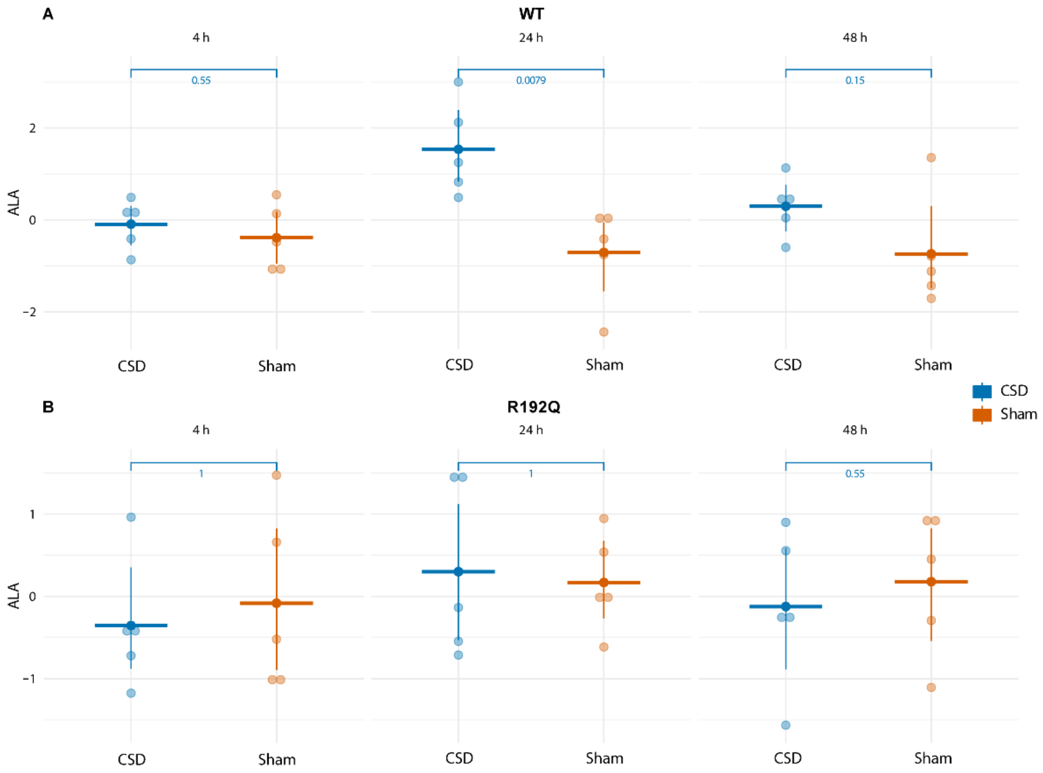

Figure S4. CSD is associated with long-term changes in ALA in WT mice. WT mice (A) showed an increase in lipid concentration of ALA at 24 h following CSD induction, that was no longer present at 48 h and not present in R192Q mutant mice (B).

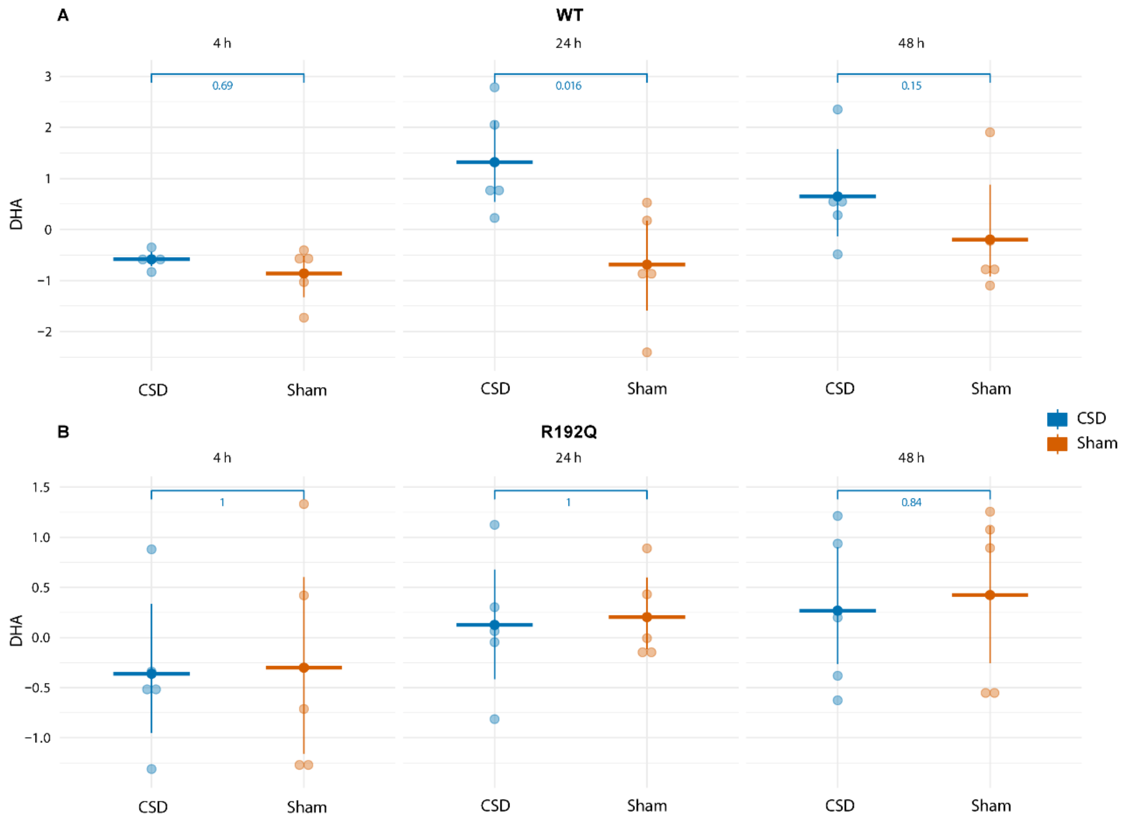

Figure S5. CSD is associated with long-term changes in DHA in WT mice. WT mice (A) showed an increase in lipid concentration of DHA at 24 h following CSD induction, that was no longer present at 48 h and not present in R192Q mutant mice (B).

Table S1. List of targeted metabolites with retention times and MS/MS transitions used for lipids quantitation.

Compound[a]	Lipid Maps ID	Retention time [min]	*m/z* in Q1	*m/z* in Q3	Declustering potential [V]	Collision energy [V]	Collision cell exit potential [V]
**Hydroxyeicosatetraenoic acids (HETEs)**							
5-HETE	LMFA03060002	8.00	319.1	115.0	-65	-18	-11
8-HETE	LMFA03060006	7.90	319.1	154.9	-70	-20	-19
11-HETE	LMFA03060003	7.90	319.1	167.0	-70	-22	-15
12-HETE	LMFA03060007	7.90	319.1	179.0	-65	-20	-23
15-HETE	LMFA03060001	7.80	319.1	219.1	-55	-18	-9
14,15-diHETE	LMFA03060077	6.95	335.1	207.0	-65	-24	-21
**Leukotrienes (LTs)**							
6-trans-LTB_4	LMFA03020013	6.65	335.1	194.9	-105	-22	-11
6-trans,12-epi-LTB_4	LMFA03020014	6.80	335.1	194.9	-80	-22	-25
LTE_4	LMFA03020002	6.95	438.1	333.1	-55	-26	-15
**Polyunsaturated fatty acids**							
AA	LMFA01030001	8.75	303.0	205.1	-155	-20	-11
DHA	LMFA01030185	8.75	327.1	229.2	-115	-18	-11
7-HDHA	n.a.	7.95	343.1	141.1	-85	-18	-23
10-HDHA	n.a.	7.90	343.1	153.0	-25	-20	-15
17-HDHA	LMFA04000072	7.85	343.1	245.0	-65	-16	-15
19,20-DiHDPA	LMFA04000043	7.35	361.1	273.0	-55	-22	-15
EPA	LMFA01030759	8.55	301.0	202.9	-125	-18	-21
LA	LMFA01030120	8.80	279.0	261.0	-115	-28	-13
ALA	LMFA01030152	8.55	277.0	233.0	-90	-22	-29
AdA	LMFA01030178	9.05	331.1	233.0	-130	-22	-11
DPAn-3	LMFA04000044	8.85	329.1	231.1	-50	-20	-17
**Prostaglandins (PGs)**							
PGD_2	LMFA03010004	4.95	351.1	233.0	-30	-16	-13
PGE_2	LMFA03010003	4.85	351.2	271.1	-50	-22	-21
8-*iso*-PGE_2	LMFA03110003	4.60	351.1	271.0	-5	-24	-19
**Thromboxanes (TX)**							
TXB_2	LMFA03030002	4.60	369.1	169.0	-55	-24	-15
**Internal standards**							
LTB_4-d_4	LMFA03020030	6.90	339.1	196.9	-70	-22	-19
15-HETE-d_8	LMFA03060080	7.80	327.2	226.0	-85	-18	-11
PGE_2-d_4	LMFA03010008	4.85	355.1	193.0	-50	-26	-17
DHA-d_5	LMFA01030762	8.75	332.0	288.1	-75	-16	-13

[a]Abbreviations: **AA**, arachidonic acid; **AdA**, adrenic acid; **ALA**, linolenic acid; **AT**, aspirin triggered; **DHA**, docosahexaenoic acid; **DiHDPA**, dihydroxydocosapentaenoic acid; **DPAn-3**, docosapentaenoic acid omega-3; **EET**, epoxyeicosatrienoic acid; **EPA**, eicosapentaenoic acid; **HDHA**, hydroxydocosahexaenoic acid; **HEPE**, hydroxyeicosapentaenoic acid; **HETE**, hydroxyeicosatetraenoic acid; **HoDE**, hydroxyoctadecadienoic acid; **HoTrE**, hydroxyoctadecatrienoic acid; ***Iso*P**, *iso*prostane; **LA**, linoleic acid; **LT**, leukotriene; **MaR**, maresin; **NeuroP**, neuroprostane; **PD**, protectins; **PG**, prostanglandin; **Q**, quadrupole; **ReV**, resolvin; **TX**, thromboxane.

[b]n.a.; information not available
